# Omission of frozen section in sentinel lymph node biopsy after neoadjuvant chemotherapy in cN0 HER2-positive and TNBC

**DOI:** 10.3389/fonc.2026.1848864

**Published:** 2026-08-18

**Authors:** Ferit Aydin, Kazim Caglar Ozcelik, Emre Tunc, Fatih Aslan, Mahmut Onur Kulturoglu, Mehmet Furkan Sagdic, Bulent Aksel, Lutfı Dogan

**Affiliations:** Department of Surgical Oncology, Ankara Etlik City Hospital, Ankara, Türkiye

**Keywords:** breast cancer, de-escalation, frozen section, HER2-positive, neoadjuvant chemotherapy, paraffin section, sentinel lymph node biopsy, triple-negative breast cancer

## Abstract

**Background:**

Axillary management in breast cancer has shifted significantly with the increasing use of neoadjuvant chemotherapy (NACT), particularly in biologically aggressive subtypes such as HER2-positive breast cancer and triple-negative breast cancer (TNBC). Intraoperative frozen section evaluation of sentinel lymph nodes (SLNs) has traditionally guided axillary dissection, but recent evidence suggests that omitting axillary dissection in patients with minimal or no residual disease provides comparable local control when combined with axillary radiotherapy. This study aimed to evaluate whether intraoperative frozen section or paraffin section assessment affects axillary management in patients who are clinically node negative (cN0) before NACT.

**Methods:**

This retrospective study included 83 patients with HER2-positive breast cancer or TNBC treated between 2023 and 2025. All patients were clinically and radiologically node negative prior to NACT. SLN biopsy was performed using isosulfan blue, dual technique, indocyanine green, or combinations. Intraoperative frozen section analysis was performed in 59 patients, while paraffin section was used in 24 patients. Clinicopathological features, SLN positivity, pathological response rates, axillary recurrence, and surgical decisions were compared. Statistical analyses were performed using SPSS 18.0, with p < 0.05 considered significant.

**Results:**

The mean age was 48.2 years. A pathological complete response (pCR) was achieved in 71% of patients, of whom 96.6% had negative SLNs. Among patients with partial response, 75% had negative SLNs. SLN positivity was observed in 8.5% (n = 5) of patients in the frozen section group and 12.5% (n = 3) in the paraffin section group. Axillary dissection was performed only in the frozen section group. No axillary recurrences occurred in either group after a median follow-up of 22 months. Paraffin section evaluation did not result in delayed treatment or increased recurrence.

**Conclusion:**

In HER2-positive and TNBC patients with cN0 status before NACT and a good pathological response, frozen section analysis of SLNs does not influence surgical decision-making. Paraffin section evaluation may represent a feasible alternative in carefully selected patients with HER2-positive breast cancer or TNBC who achieve an excellent response to neoadjuvant chemotherapy. However, these findings should be interpreted cautiously given the retrospective design, small sample size, and relatively short follow-up, and require validation in larger prospective studies.

## 1 Introduction

Surgical management of the axilla in breast cancer has evolved substantially following the introduction of neoadjuvant chemotherapy (NACT). Although the detection of lymph node metastasis—or even isolated tumor cells using frozen sections after NACT was once considered an indication for axillary dissection, current evidence demonstrates that omitting axillary dissection in patients with minimal residual disease yields comparable rates of axillary recurrence when combined with axillary radiotherapy (1).This finding implies that intraoperative frozen section evaluation of sentinel lymph nodes (SLNs) may not alter surgical decision-making in selected patients. In particular, for patients who are clinically node negative (cN0) prior to NACT and remain without clinical or radiologic suspicion of axillary involvement after treatment, the utility of frozen section analysis is limited, as it does not significantly influence the surgical approach. Furthermore, awaiting intraoperative pathology results prolongs operative time and anesthesia exposure, increases healthcare costs, and adds to the surgical workload (2).

In this study, we investigated whether intraoperative frozen section or paraffin section assessment of SLNs affects the decision of axillary dissection in patients with biologically aggressive tumors, such as HER2-positive breast cancer and triple-negative breast cancer (TNBC), who were clinically node-negative (cN0) prior to NACT. We also aimed to identify the subgroup of patients in whom SLN biopsy could safely be evaluated with paraffin section instead of frozen section.

## 2 Materials and methods

Data from 83 patients with HER2-positive breast cancer or TNBC treated between 2023 and 2025 were retrospectively analyzed. Eligible patients were cN0 upon physical examination; those who were cN0 but had suspicious lymph nodes upon imaging with biopsy-proven benign cytology results were also included. Patients who were clinically node-positive (cN+) before NACT or who had biopsy-proven malignant lymph nodes upon imaging were excluded. Prior to NACT, all patients underwent axillary evaluation with ultrasonography and positron emission tomography. Treatment decisions were made by a multidisciplinary tumor board including medical oncology, surgical oncology and radiation oncology specialists. The therapeutic regimen consisted of an adriamycin–taxane-based neoadjuvant chemotherapy protocol, with dual HER2 blockade added for patients with HER2-positive breast cancer. Following NACT, all patients underwent another axillary evaluation with ultrasonography. The study was approved by the Ethics Committee of Ankara Etlik City Hospital (Approval No: AEŞ-BADEK2-2025-157). Informed consent to participate was obtained from all participants. This study was conducted in accordance with the principles of the Declaration of Helsinki.

SLN mapping was performed using isosulfan blue dye (Lymphazurin™, Covidien, Mansfield, MA, USA), dual technique with gamma probe (Neoprobe Gamma Detection System, Devicor Medical Products, Cincinnati, OH, USA), indocyanine green (ICG, Daiichi Sankyo, Tokyo, Japan), or a combination of these methods. In this retrospective analysis, lymph node frozen section examination was performed in 59 patients, whereas paraffin section evaluation was carried out in 24 patients comprising the study arm. The choice between frozen section and paraffin section was not randomized. Instead, it reflected the evolution of institutional practice over the study period. Patients treated earlier in the study period more commonly underwent frozen section analysis, whereas paraffin section evaluation was increasingly adopted following implementation of our institutional axillary de-escalation strategy. Therefore, the selection of the pathological assessment method was primarily time-dependent rather than based on tumor biology or patient characteristics. In patients who underwent frozen section analysis, axillary dissection was performed when macrometastasis, micrometastasis, or isolated tumor cells were detected in the lymph node. In contrast, in the paraffin section group, the decision for axillary dissection was made by the tumor board based on the number of metastatic lymph nodes indicated in the final pathology report.

All pathological evaluations were performed by board-certified breast pathologists with at least 5 years of experience, and the same pathology team evaluated both frozen and paraffin sections to ensure consistency. In patients with HER2-positive disease who achieved a pathologic complete response, HER2 blockade therapy was continued to complete one year. Those with a partial response received trastuzumab emtansine (T-DM1). Adjuvant radiotherapy was delivered according to institutional and contemporary guideline recommendations. Patients undergoing breast-conserving surgery received whole-breast irradiation with regional nodal irradiation when indicated, whereas patients undergoing mastectomy received chest wall irradiation with or without regional nodal irradiation according to pathological nodal status and multidisciplinary tumor board recommendations. Radiation therapy was administered at a total dose of 50 Gy in 25 fractions. All patients were followed every 3 months for the first two years and every 6 months thereafter, with routine complete blood count, tumor markers, and breast and axillary ultrasonography. All data were statistically analyzed using SPSS Statistics for Windows, Version 18.0 (SPSS Inc., Chicago, IL, USA). As this was a retrospective study including all eligible patients treated within the study period, no formal sample size calculation was performed. Continuous variables were tested for normality using the Shapiro–Wilk test. Normally distributed data were compared using the Student’s t-test, while non-normally distributed data were analyzed with the Mann–Whitney U test. Categorical variables were compared using the Chi-square test or Fisher’s exact test, as appropriate. A p-value <0.05 was considered statistically significant.

## 3 Results

The mean patient age was 48.2 years (range: 32–69 years). The mean clinical tumor size was 25.3 mm (range: 10–58 mm), with 24 (28.9%), 55 (66.3%), and 4 (4.8%) patients having T1, T2, and T3 tumors, respectively. Moreover, 39 patients (47.0%) were hormone receptor–positive HER2-positive, 25 (30.1%) were hormone receptor–negative HER2-positive, and 19 (22.9%) had TNBC. On pre-NACT ultrasonography, 85.5% of patients had reactive lymph nodes, and 14.5% (n = 12) had suspicious nodes, with benign cytology confirmed with fine-needle aspiration biopsy. Following NACT, 59 patients (71%) achieved a pathological complete response (pCR) in the primary tumor, and among these, 57 patients (96.6%) had no sentinel lymph node metastasis. Among the 24 patients with a partial response, 18 patients (75%) had negative SLNs. (**Table 1**).

**Table 1:** Correlation between clinical response to neoadjuvant chemotherapy and sentinel lymph node biopsy results.

Clinical response to NACT	Intraoperative pathological examination	SLNB result	Count	% within intraoperative examination	Total
Partial response	Performed	Negative	14	73.7%	
		Positive	5	26.3%	
		Total	19	100.0%	19
	Not performed	Negative	4	80.0%	
		Positive	1	20.0%	
		Total	5	100.0%	5
	Overall Total		24	100.0%	24
Complete response	Performed	Negative	40	95.2%	
		Positive	2	4.8%	
		Total	42	100.0%	42
	Not performed	Negative	17	100.0%	
		Positive	0	0.0%	
		Total	17	100.0%	17
	Overall Total		59	100.0%	59

NACT, neoadjuvant chemotherapy; SLN, sentinel lymph node; SLNB, sentinel lymph node biopsy.

Data are presented as number (percentage). Percentages represent row-wise distributions within intraoperative pathological examination groups. Statistical comparisons were performed using the Chi-square test or Fisher’s exact test, where appropriate. A p-value < 0.05 was considered statistically significant.

Breast-conserving surgery was performed in 62 patients (74.7%) of patients, whereas 12 patients (14.5%) underwent simple mastectomy. In addition, five patients with BRCA mutations underwent bilateral nipple-sparing mastectomy with silicone implant reconstruction, and four patients underwent unilateral nipple-sparing mastectomy with silicone implant reconstruction.

SLN biopsy was performed using isosulfan blue dye alone in 48.2% of patients, combined techniques in 41%, indocyanine green in two patients, and the dual method alone in seven patients. The mean number of dissected SLNs was 4 (range: 2–7). Frozen section analysis was performed in 59 patients (71.1%), whereas paraffin section analysis was performed in 24 (28.9%). The clinical and histopathological features of both groups were comparable (**Table 2**).

**Table 2:** Clinicopathological characteristics of patients according to the method of sentinel lymph node evaluation.

Clinicopathological characteristics	Frozen section % (n=59)	Paraffin section % (n=24)	OR (95% CI)	P-value
Age				
<45 years	45.8% (27)	33.3% (8)	0.59 (0.22–1.60)	0.33
≥45 years	54.2% (32)	66.7% (16)		
Hormone receptor status				
HR-positive/HER2+	45.8% (27)	50.0% (12)	–	0.44
HER2+	33.9% (20)	20.8% (5)		
TNBC	20.3% (12)	29.2% (7)		
Pre-NACT USG				
Reactive	88.1% (52)	79.2% (19)	1.95 (0.553-6.91)	0.31
Suspicious	11.9% (7)	20.8% (5)		
Post-NACT USG				
Reactive	96.6% (57)	95.8% (23)	1.24 (0.107-14.3)	1.00
Suspicious	3.4% (2)	4.2% (1)		
Response to NACT Complete response	69.5% (41)	75.0% (18)	1.32 (0.448-3.87)	0.82
Partial response	30.5% (18)	25.0% (6)		

NACT, neoadjuvant chemotherapy; USG, ultrasonography; HR, hormone receptor; HER2, human epidermal growth factor receptor 2; TNBC, triple-negative breast cancer; SLN, sentinel lymph node.

Data are presented as number (percentage). Percentages are calculated within each column. Comparisons between groups were performed using the Chi-square test or Fisher’s exact test, as appropriate. A p-value < 0.05 was considered statistically significant.

In the paraffin section group, the mean number of dissected SLNs was 4 (range: 2–7). None of the patients had grossly metastatic lymph nodes intraoperatively. Final pathology revealed only one micrometastatic lymph node in three patients (12.5%); however, complete axillary dissection was not performed. During a mean follow-up of 22 months, no axillary recurrences were observed in this group.

In the frozen section group, the mean number of dissected SLNs was also 4 (range: 2–8). Lymph node metastasis was detected in five patients (8.5%), who subsequently underwent axillary dissection, which yielded a mean of 13 (range: 10–18) nodes. Among the five patients in the frozen section group with lymph node metastasis, only a single metastatic lymph node was identified in each case. Immunohistochemical evaluation revealed a macrometastasis with capsular invasion in one patient; axillary dissection in this patient identified only one additional non-sentinel metastatic lymph node. In the remaining four patients, immunohistochemical analysis demonstrated micrometastases, and no additional metastatic lymph nodes were detected in the axillary dissection specimens. During a mean follow-up of 22 months, no axillary recurrences were observed in this group.

## 4 Discussion

Pre-NACT clinical lymph node status remains critical in determining the surgical management of the axilla. Landmark trials, such as ACOSOG Z1071 and SENTINA, have demonstrated that in patients with initially node-positive (cN+) disease who achieved a good response to NACT, SLN biopsy could safely replace axillary dissection, thereby altering the surgical approach to the axilla (3, 4). However, these studies recommended axillary dissection when isolated tumor cells or micrometastases were present in the SLN.

Subsequent studies have challenged this approach, concluding that performing only SLN biopsy and avoiding axillary dissection in patients with a good response to NACT and residual metastatic lymph nodes can yield comparable oncologic outcomes when axillary radiotherapy is administered. The OPBC-05/ICARO study reported that patients with isolated tumor cells detected in the SLN after NACT had similar recurrence rates whether treated with axillary dissection or axillary radiotherapy alone (5). Similarly, the combined analysis of Neosenti-Turk MF-18-02/18–03 revealed that in patients with cN1–2 disease prior to NACT, axillary radiotherapy without axillary dissection achieved comparable results not only in cases with isolated tumor cells/micrometastases but also in those with macrometastases (6). Müslümanoğlu et al. observed that SLN biopsy combined with axillary radiotherapy was sufficient in patients with cN1 disease with good pathological response (>50% fibrosis) and <3 positive lymph nodes, instead of performing axillary dissection (1).

In our study, only a single metastatic lymph node was identified in three patients who underwent paraffin section evaluation. These patients were assessed by the multidisciplinary tumor board based on the number of excised sentinel lymph nodes, the number of metastatic lymph nodes, and overall tumor burden, and axillary dissection was not performed. However, if intraoperative frozen section had been performed in these patients, it is likely that a decision for axillary dissection would have been made, which may have resulted in over treatment in light of current evidence. Therefore, the use of intraoperative frozen section in cN0 patients who achieve a good response to neoadjuvant therapy may be associated with unnecessary axillary dissection.

Supporting this observation, in our study, five patients in the frozen section group were found to have a single metastatic lymph node each and subsequently underwent axillary dissection. Examination of the axillary dissection specimens revealed an additional non-sentinel lymph node metastasis in only one patient, while no metastatic lymph nodes were identified in the remaining four patients. This finding raises the question of whether axillary dissection may have been unnecessary in this subset of patients.

These studies suggest that axillary dissection may be omitted even in patients with positive axillary lymph node metastasis before neoadjuvant chemotherapy when a good treatment response is achieved in metastatic lymph nodes. Therefore, identifying a positive sentinel lymph node by frozen section does not change the decision for axillary dissection in some patients. This finding raises the question of whether paraffin section evaluation of sentinel lymph nodes could be used instead of frozen section in selected patients.

In patients with TNBC and HER2-positive breast cancer, the rate of pathological complete response (pCR) following neoadjuvant chemotherapy (NACT) is approximately 40%–50%. In this patient population, pCR rates are higher compared to those with luminal A and B subtypes (7, 8). Furthermore, NACT is also effective in eradicating lymph node metastases (9–11). When pCR is achieved in the primary tumor, negative axillary nodes or axillary pCR is expected. In a study conducted at MD Anderson, of 290 cN0 patients with HER2-positive breast cancer or TNBC, 40.4% achieved pCR in the breast, and none of these patients had axillary metastasis (12). In the same study, among 237 biopsy-proven cN+ patients, pCR was achieved in the breast in 77 patients, of which 89.6% had no residual axillary metastasis. Furthermore, of 160 patients without breast pCR, 42.5% were free of axillary metastasis.

In our study, among patients who were cN0 before NACT, 71% achieved pCR in the primary tumor, of whom 96.6% had negative SLNs. Even among patients with partial response, 75% had negative SLNs. These findings suggest that in patients with HER2-positive breast cancer and TNBC who are cN0 before NACT, and in this subgroup, paraffin section evaluation may safely replace intraoperative frozen section for SLN evaluation. Our findings are supported by a database study of patients with 6802 cN0 HER2-positive breast cancer and 6222 cN0 TNBC with pCR rates of 45% and 37%, respectively. In patients achieving pCR, the rate of axillary metastasis after NACT was only 1.6% in both subgroups, implying that axillary surgery could be avoided for these patients (13).

Despite these findings, variations in axillary management persist among surgeons. A national survey of 101 surgeons in Türkiye revealed that 76% always performed intraoperative frozen section, whereas only 5% did not (14). Notably, 46.5% of surgeons did not perform axillary dissection in cN0 patients when 2–3 SLNs were retrieved and ≤2 nodes harbored micrometastases or macrometastases. For these surgeons, frozen section analysis of SLNs did not influence their surgical decision-making.

Several limitations should be acknowledged. First, this was a retrospective single-center study with a relatively limited sample size, particularly in the paraffin section group. Second, the median follow-up of 22 months is insufficient to draw definitive conclusions regarding long-term axillary recurrence and oncologic safety. Third, the choice between frozen and paraffin section was not randomized and reflected changes in institutional practice over time, introducing potential selection bias. Finally, treatment pathways differed between the two groups. Patients with positive frozen section findings generally underwent immediate ALND, whereas management in the paraffin section group was determined after multidisciplinary tumor board review according to evolving de-escalation principles. This difference should be considered when interpreting the comparative outcomes. Larger prospective multicenter studies with longer follow-up are warranted to validate our findings and more definitively assess the role of paraffin section evaluation in this setting.

In patients with HER2-positive breast cancer and TNBC who are clinically and radiologically node-negative before NACT and achieve a favorable pathological response, intraoperative frozen section analysis of sentinel lymph nodes may have a limited impact on surgical decision-making. In this selected subgroup, paraffin section evaluation may represent a feasible alternative to routine frozen section analysis. However, given the retrospective single-center design, relatively small sample size, and limited follow-up, these findings should be interpreted with caution. Larger prospective multicenter studies are needed to validate these results and to establish standardized criteria for identifying patients who may be suitable for paraffin section evaluation alone.

## Data Availability

The data analyzed in this study is subject to the following licenses/restrictions: The data analyzed in this study is subject to the following licenses/restrictions: Restrictions apply to the availability of these data due to ethical and legal considerations related to patient privacy. The datasets generated and/or analyzed during the current study are not publicly available but are available from the corresponding author on reasonable request, subject to approval by the institutional ethics committee. Requests to access these datasets should be directed to MS (e-mail: m.f.sagdic@gmail.com). Requests to access these datasets should be directed to m.f.sagdic@gmail.com.
